# Tubercular Peritonitis, &c.

**Published:** 1894-03-10

**Authors:** 


### TUBERCULAR PERITONITIS, &c.

It has long been an acknowledged fact that cases of tubercular peritonitis recover spontaneously, especially those in which there has been considerable exudation, and a large number are now on record in which laparotomy has been followed by encouraging results. Two interesting papers recently published on the subject, one by Dr. Conitzer[1] and the other by Dr. Nolen[2], may thus be summarised[3]. The former reports seven cases, all occurring in children, in which laparotomy had been performed, the ages varying from two to nine years. In four of the cases there was a considerable amount of fluid in the abdominal cavity, and the peritoneum itself exhibited numerous small tubercles on its parietal and visceral surfaces. The general health of these children was fairly good. In the other three cases there was no fluid, but the constitutional disturbance was greater, and the intestines and omentum were matted together.

The operation consisted of an incision through the abdominal parietes, allowing the fluid to escape, and, without further interference of any kind, closing up the wound. In all the cases the diagnosis was confirmed by microscopical examination. The four cases in which there was effusion all did well. Of the other three patients two died shortly after the operation without relief, but the condition of the third patient was decidedly improved. Dr. Conitzer considers, therefore, that all forms of tubercular peritonitis may be relieved by operation, even when simple puncture has failed, but the best results are those obtained in cases in which effusion has taken place. He would not advise operation in advanced cases, or in those in which there is marked tuberculosis of other organs. Dr. Nolen considers that the reason why cases of tubercular peritonitis do so well after laparotomy is because air is admitted to the abdominal cavity, and that consequently an irritative process is started, in the course of which the bacilli become surrounded by a capsule, which gradually becomes cretified, so destroying the germs and their products. Acting on this theory, he does not believe laparotomy, to be necessary, but simply performs paracentesis, removes as much of the fluid as possible. and then injects air into the peritoneal cavity. The air is rendered completely dry and aseptic by passing it through suitable apparatus. He has tried the treatment in two cases. The first was a typical case of tubercular peritonitis; the patient was very ill and the prognosis was very grave. After withdrawing the fluid, air was forced in and the abdomen gently kneaded so as to bring the air, as far as possible, into contact with the whole of the peritoneum. A light bandage was then applied and most of the air was driven out by pressure. After the operation the patient had a slight increase of peritonitis, but afterwards did well and was discharged convalescent. The second case was complicated with pulmonary tuberculosis, and by an umbilical hernia. The same method of procedure was adopted, and with the same success so far as the peritonitis was concerned. Dr. Nolen thinks this treatment is worthy of further trial. Dr. Poncet, after performing laparotomy, dusts three to four grammes of iodoform over the intestines before closing the abdominal wound. A successful case treated in this way is reported.[4]

Mr. Howard Marsh says[5] that three main conditions have been found in tubercular peritonitis: (*a*) In some cases a considerable amount of fluid has been present, clear, turbid, or purulent, and mixed with more or less caseous material; (*b*) sometimes the fluid instead of being free is walled in by thick and firm adhesions, so that a clearly defined, encysted and movable tumour was present; (*c*) no fluid is present, the inflammation being plastic, and leading to matting of the intestines together, and sometimes producing intestinal obstruction. He thinks operation advisable in groups *a* and *b*, but not in *c*, although several cases are on record in which, on a mistaken diagnosis, laparotomy has been performed, tuberculous matting found, the wound thereupon closed, and the patient has at once improved and ultimately recovered. When the fluid is purulent, the removal of pus may be followed by an uninterrupted recovery. With reference to the remote results of laparotomy practised for the cure of local peritonitis, Pick, after having collected one hundred and thirty cases of tubercular peritonitis so treated, previous to 1890, states[6] that intervention is useless in acute miliary peritonitis. In the dry ulcerative form of the disease no cures are reported; in the suppurative ulcerative form nearly forty-three per cent. are cured; in the dry fibrous form over seventy-one per cent. are cured; in the generalized ascitic form over seventy-three per cent. are cured; in the encysted ascitic form over ninety per cent. are cured. Beaussenat examined five of the cases found in Pick's tables several years after the operation was performed, and in all found that cure was permanent. He admits, too, that in cases of generalized fibrous ascites laparotomy gives in the adult about seventy-two per cent. of cures, among which about fifty-three per cent. will probably be durable. There are three contra-indications to intervention—high fever, extensive or rapid pulmonary or pleural tuberculosis, and tubercular enteritis. The abdominal incision is an essential element in obtaining curative results. Drainage should not be employed.

[1] Deut. Med. Woch., July 20th, 1893, and Brit. Med. Journ., Aug. 12th, 1893. [2] Berl. Klin. Woch., Aug. 21st, 1893, and Brit. Med. Journ., Sept. 16th, 1893. [3] Brit. Med. Journ., Oct. 14th, 1893. [4] Annals of Surgery, May, 1893. [5] Brit. Med. Journ., Sept. 30th, 1893. [6] L'Union Medicale, 47 année, No. 36, March, 1893, and Therapeutic Gazette, May 18th, 1893.

